# Whole-Brain Monosynaptic Afferents to Rostromedial Tegmental Nucleus Gamma-Aminobutyric Acid-Releasing Neurons in Mice

**DOI:** 10.3389/fnins.2022.914300

**Published:** 2022-06-06

**Authors:** Ya-Nan Zhao, Yang Zhang, Shi-Yuan Tao, Zhi-Li Huang, Wei-Min Qu, Su-Rong Yang

**Affiliations:** Department of Pharmacology, School of Basic Medical Sciences, State Key Laboratory of Medical Neurobiology and MOE Frontiers Center for Brain Science, Institutes of Brain Science, Fudan University, Shanghai, China

**Keywords:** rostromedial tegmental area (RMTg), GABAergic neurons, mouse, rabies virus retrograde tracing, monosynaptic inputs

## Abstract

Increasing evidence has revealed that the rostromedial tegmental area (RMTg) mediates many behaviors, including sleep and addiction. However, presynaptic patterns governing the activity of γ-aminobutyric acid-releasing (GABAergic) neurons, the main neuronal type in the RMTg, have not been defined. Here, we used cell-type-specific retrograde trans-synaptic rabies viruses to map and quantify the monosynaptic afferents to RMTg GABAergic neurons in mouse whole brains. We identified 71 ascending projection brain regions. Sixty-eight percent of the input neurons arise from the ipsilateral and 32% from the contralateral areas of the brain. The first three strongest projection regions were the ipsilateral lateral hypothalamus, zone incerta, and contralateral pontine reticular nucleus. Immunohistochemistry imaging showed that the input neurons in the dorsal raphe, laterodorsal tegmentum, and dorsal part of zone incerta were colocalized with serotoninergic, cholinergic, and neuronal nitric oxide synthetase-expressing neurons, respectively. However, in the lateral hypothalamus, a few input neurons innervating RMTg GABAergic neurons colocalized orexinergic neurons but lacked colocalization of melanin-concentrating hormone neurons. Our findings provide anatomical evidence to understand how RMTg GABAergic neurons integrate diverse information to exert varied functions.

## Introduction

The rostromedial tegmental area (RMTg) was first defined in rats. When rats are administered psychostimulants, such as cocaine, a large number of neurons in the RMTg express the immediate-early gene product c-Fos (Geisler et al., 2008). It has been demonstrated that the RMTg is primarily innervated by the lateral habenula (LHb) in the thalamus (Herkenham and Nauta, 1979; Jhou et al., 2009b) and sends particularly dense projections to the ventral tegmental nucleus (VTA) or substantia nigra compacta (SNc), and the dorsal raphe nucleus (DR) (Sego et al., 2014; Metzger et al., 2019). Thus, the RMTg has been proposed to be implicated in the aversive emotion process (Glover et al., 2016; Fu et al., 2017). However, increasing evidence has illustrated that the RMTg plays crucial roles in many other regulatory functions, such as sleep-wake regulation (Yang S. R. et al., 2018), depression (Proulx et al., 2018; Elmer et al., 2019), and substance abuse disorders including opioid and alcohol addictions (Zhao et al., 2020). Li et al. (2019b) recently reported that the RMTg drives triply dissociable responses to aversive cues, outcomes, and prediction errors from the prelimbic cortex, brainstem parabrachial nucleus (PB), and LHb, respectively. These studies showed that the heterogeneous functions of the RMTg are likely mediated by multiple afferents. Conventionally, fluorogold and cholera toxin subunit b (CTb) are two widely utilized retrograde tracers in tracing studies. Traditional tracing approaches provide important classifications of major afferent inputs; however, traditional tracing cannot distinguish between cell types. Moreover, the traditional tracers are less efficient. There is a decrease in fluorescent intensity or the number of labeled cells with time (Dederen et al., 1994; Novikova et al., 1997). Although the RMTg is primarily composed of γ-aminobutyric acid-releasing (GABAergic) neurons, they account for about 70–92% of the total neurons (Jhou et al., 2009a). Previous anatomical and functional studies of the RMTg were mainly conducted in rats; however, the common use of transgenic mice, together with optogenetics and chemogenetics, has made it possible to manipulate specific subtypes of certain nuclei. In addition, most recent functional studies of the RMTg have been performed in mice (Li et al., 2019b; Taylor et al., 2019; Markovic et al., 2021; Vlasov et al., 2021).

Recently, the development of the genetically modified retrograde trans-synaptic rabies virus (RV) has allowed us to identify the whole-brain presynaptic input neurons in a specific neuronal population within a complicated neural network. In combination with immunohistochemistry, neuronal types of specific inputs can be further characterized (Yuan et al., 2018; Xu X. et al., 2020). To overcome the limitations of traditional tracing technology, we utilized the viral tracing method to map the whole-brain monosynaptic afferent inputs to RMTg GABAergic neurons in mice. We then labeled several molecular markers related to sleep-wake regulation and compared the statistical differences between ipsilateral and contralateral inputs. Our findings provide a structural framework for understanding the diverse physiological functions of RMTg GABAergic neurons.

## Materials and Methods

### Animals

All protocols were approved by the Committee on the Ethics of Animal Experiments of the School of Basic Medical Sciences, Fudan University, China, with the license identification number 20210302-105. Male and female vesicular GABA transporter (VGAT)-Cre mice (Jackson Laboratory stock 017535) at 8–10 weeks old were used in this study (Chao et al., 2010). Mice were housed in a 12-h light-dark cycle (lights on at 07:00 and off at 19:00, illumination intensity approximately 100 lx) at ambient temperature (22 ± 0.5°C), and with a relative humidity of 60 ± 2%. Food and water were provided *ad libitum* (Yan et al., 2021).

### Viruses and Viral Injections

All viruses used in this study were purchased from BrainVTA (Wuhan, China). Mice were anesthetized with 1.5–2% isoflurane and placed on a stereotaxic apparatus. After asepsis, the skin was cut to expose the skull, and the overlying connective tissue was removed. A small craniotomy was performed above the superficial layer of the RMTg. A 20-nL mixture of the help viruses of AAV (adeno-associated virus)–EF1α-DIO-H2B-eGFP-T2A-TVA (tumor virus receptor A) and AAV-EF1α-DIO-RG (rabies glycoprotein) was slowly injected (40 nL/min) into one side of the RMTg. The mice were surgically sutured and kept on a heating pad until they awoke from the anesthesia. Two weeks later, 50 nL of EnvA-RG-deleted and dsRed-expressing RV (RV-EnvA-ΔG-dsRed) was microinjected in the same site. The viral injection coordinates in the RMTg refer to the bregma −3.8 mm, 0.5 mm from midline, and at a 4.0 mm depth from the dura. Considering the valid viral infection and neurotoxicity of RV, the mice were perfused one week later after RV delivery (Ciabatti et al., 2017; Chen et al., 2022).

### Immunohistochemistry

After being deeply anesthetized, mice were transcardially perfused with phosphate-buffered saline (PBS), followed by 4% paraformaldehyde in PBS. For fixation, brains were kept overnight in 4% paraformaldehyde. Each brain was placed in 30% sucrose in PBS for approximately 48 h. After embedding and freezing, the brains were sectioned into 30 μm coronal slices using a microtome (CM1950, Leica, Germany).

For the immunofluorescence assay, brain slices were washed three times using PBS and then incubated with primary antibodies [rabbit anti-Foxp1, 1:20,000, ab16645, Abcam, Waltham, MA, United States (Lahti et al., 2016; Smith et al., 2019); goat anti-choline acetyltransferase (ChAT), 1:1000, AB144P, Millipore, Sheboygan Falls, WI, United States (Van Dort et al., 2015); goat anti-hypocretin A (Hcrt), 1:600, sc-80263, Santa Cruz Biotechnology, Dallas, TX, United States (Li et al., 2022); rabbit anti-melanin concentrating hormone (MCH), 1:1000, H-070-47, Phoenix Pharmaceuticals, Burlingame, CA, United States (Apergis-Schoute et al., 2015); rabbit anti-serotonin (Sero), 1:5000, S5545, Sigma, St. Louis, MO, United States (Baum et al., 2018); goat anti-parvalbumin (PV), 1:1000, PVG213, Swant, Burgdorf, Switzerland (Schwaller et al., 1999); rabbit anti-neuronal nitric oxide synthase (nNOS), 1:200, 61-7000, Thermo Fisher, Waltham, MA, United States (Ding et al., 2005; Li and Spitzer, 2020)] dissolved in PBS-Triton (0.3% Triton X-100 in PBS) overnight at 4°C. The next day, slices were washed with PBS and incubated with Alexa Fluor 488-conjugated IgG secondary antibodies (donkey anti-rabbit, 1:1000; Jackson ImmunoResearch, United States; donkey anti-goat, 1:1000, Jackson ImmunoResearch, United States) for 2 h at room temperature. The immunohistochemical reaction was terminated by washing three times with PBS. Next, the sections were stained for nuclei with 4’, 6-diamidino-2-phenylindole (DAPI, 1:10000, D9542, Sigma-Aldrich, United States) for 10 min. Finally, the sections were mounted on glass slides and cover-slipped with Fluoromount G™ (Southern Biotech, Birmingham, AL, United States) for imaging.

### Imaging and Data Analysis

For whole-brain retrograde tracing, one out of every four sections was captured using a 20 × objective on a microscope (VS-120, Olympus, Tokyo, Japan). Other fluorescence images were captured using a confocal microscope (Nikon AIR-MP). Each slice was matched to the corresponding atlas level of the mouse brain atlas (Franklin and Paxinos, 2001). The dsRed-expressing neurons in individual nuclei within each whole brain were quantified semi-automatically using ImageJ software. First, the images were converted into 8-bit grayscale and adjusted using a threshold. Before automatic quantification with analysis of particles, cell size and circularity were set accordingly. Next, dsRed-labeled cells were manually checked. The proportion of input neurons from each of the 71 brain regions was calculated as the ratio of the total number of dsRed-labeled cells in each brain (including both sides with the injection site excluded). The neurons in each brain region, with a proportion of more than 0.1% of the total monosynaptic inputs, were calculated. Based on the proportion of dsRed-labeled cells in each nucleus, we defined the following four grades of afferent inputs: numerous inputs (over 6%), large inputs (3–6%), moderate inputs (1–3%), and few inputs (<1%), as shown in Figures 6, 7. All data are presented as mean ± standard error of the mean.

## Results

### Mapping Monosynaptic Inputs to Rostromedial Tegmental Area GABAergic Neurons Using the Rabies Virus-Mediated Trans-Synaptic Tracing System

To identify the monosynaptic afferents to RMTg GABAergic neurons, we used RV-mediated, trans-synaptic retrograde tracing on a transgenic mouse line expressing Cre recombinase in GABAergic neurons. This system has been shown to efficiently label monosynaptic inputs to specifically infected starter cells (Wickersham et al., 2007).

**FIGURE 1:**
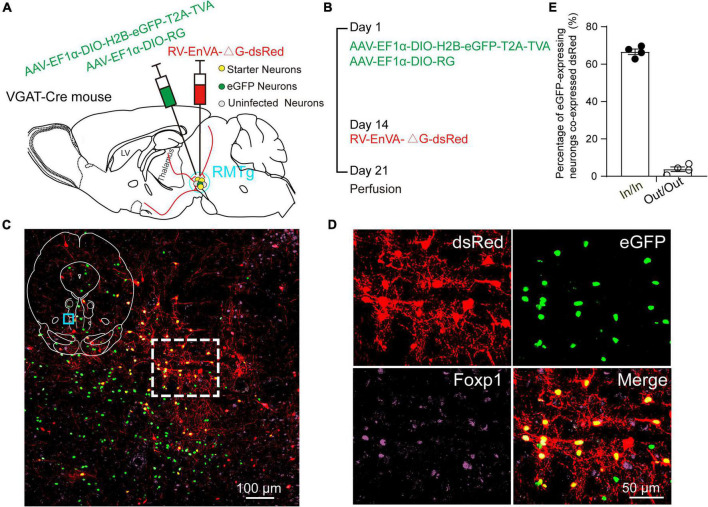
Virus injection and starter neurons in the rostromedial tegmental area (RMTg) in the vesicular GABA transporter (VGAT)-Cre mice. **(A)** Schematic diagram of injection of helper viruses, including adeno-associated virus (AAV) expressing tumor virus receptor A (TVA) with a green fluorescent protein (AAV-EF1α-DIO-H2B-eGFP-T2A-TVA) or expressing rabies glycoprotein (RG) (AAV-EF1α-DIO-RG) into the RMTg in a VGAT-Cre mouse, followed by injection of modified rabies virus (RV) expressing dsRed (RV-EnVA-△G-dsRed). **(B)** Experimental timeline for injection of helper viruses, RV, and perfusion. **(C)** Immunostaining showed the presence of merged neurons (yellow) in the RMTg. The dashed square region is the magnification of the indigo square region in the left upper islet. **(D)** Higher magnification of the dashed square region in C. Red, RV-infected neurons; green, helper virus-infected neurons with no RV infection; purple, neurons stained with Forkhead box protein 1 (Foxp1); yellow, starter neurons merged with both helper viruses and RV. **(E)** Co-expression rate of dsRed-labeled neurons in eGFP-expressing neurons. In, inside the RMTg; Out, outside the RMTg. *n* = 4, each data point represents one experimental mouse.

Using VGAT-Cre mice, the avian TVA and the RG were first expressed in RMTg GABAergic neurons achieved by unilateral injection of two kinds of helper viruses (AAV-EF1α-DIO-H2B-eGFP-T2A-TVA and AAV- EF1α-DIO-RG) into the RMTg. After 2 weeks, glycoprotein (G) gene-deleted RVs expressing dsRed (RV-EnVA-△G-dsRed) were injected into the same site, where the RVs only infected TVA-expressing cells and required RG expression to spread retrogradely into presynaptic cells (Figures 1A,B). The starter neurons, which are shown in yellow, were characterized by the expression of both the helper virus and RV. We found that 67% of the eGFP-expressing neurons co-expressed RV within the RMTg region, but only about 4% in the adjacent area of the RMTg. The results indicated that the dsRed-expression of the RV primarily limited to the RMTg region. The starter neurons were shown to co-express Foxp1, which is recognized as a molecular marker of the RMTg. There were some neurons infected only with RV but not the helper virus in the RMTg, indicating the existence of local monosynaptic inputs to RMTg GABAergic neurons (Figures 1C–E).

**FIGURE 2:**
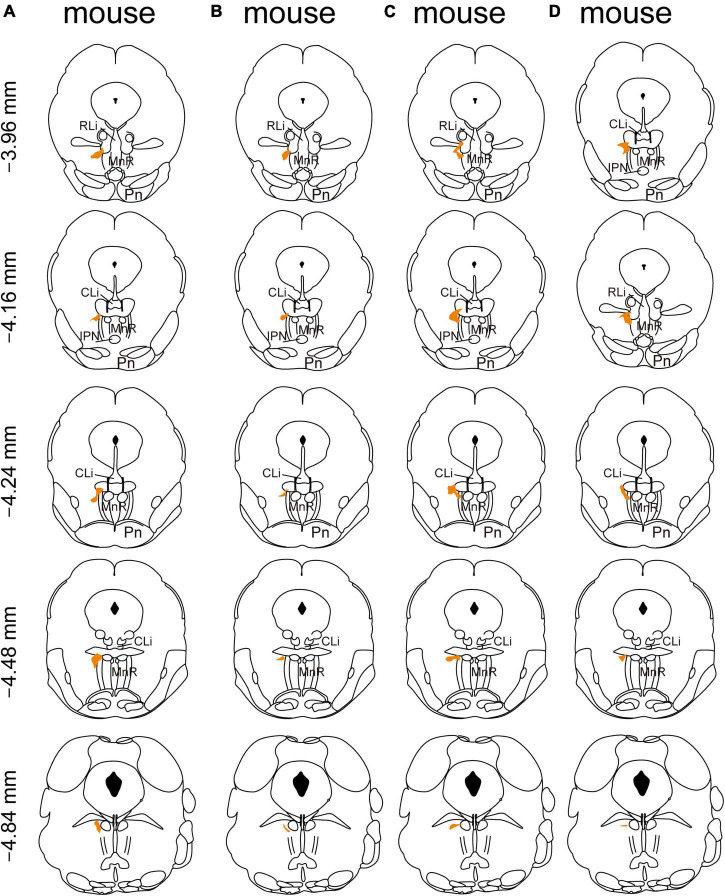
Mapping of the starter neurons in the rostromedial tegmental area (RMTg) in vesicular GABA transporter (VGAT)-Cre mice **(A–D)**. Coronal sections showing the distribution of starter neurons in the RMTg (between −3.96 mm and −4.84 mm from bregma) of four VGAT-Cre mice. The area of starter neurons is depicted by the orange shaded region.

In addition, we mapped the starter neurons in the RMTg from five brain slice levels for each of the four mice and showed that they were mostly limited to the RMTg. The starter neurons were mainly observed in the coronal planes between −3.96 and −4.84 mm from the bregma (Figure 2).

### Overview of Input Patterns to Rostromedial Tegmental Area GABAergic Neurons

**FIGURE 3:**
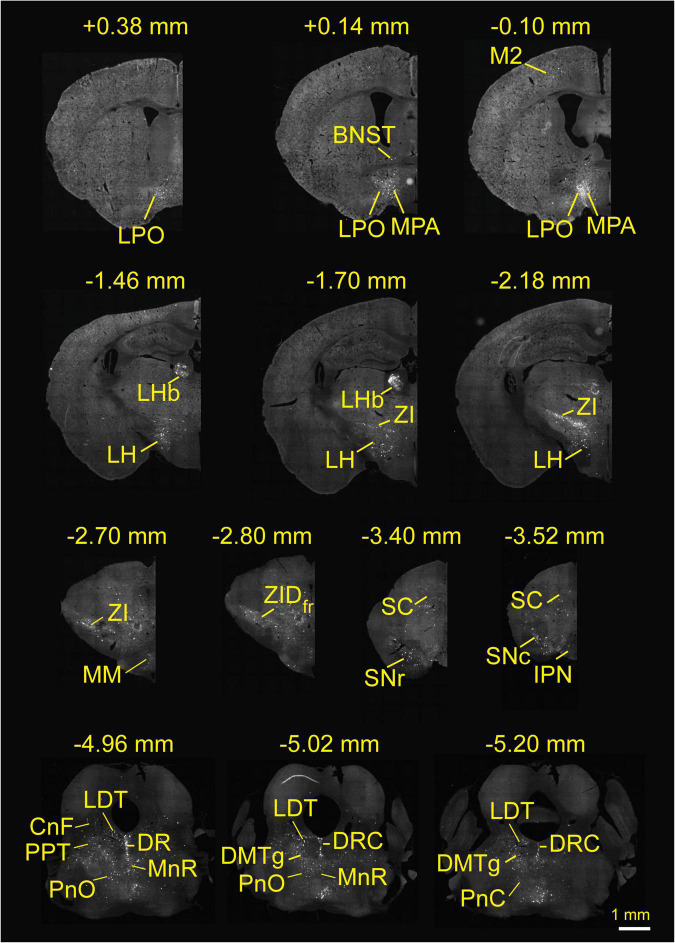
Whole-brain monosynaptic inputs to rostromedial tegmental area (RMTg) γ-aminobutyric acid (GABA)-releasing neurons in vesicular GABA transporter-Cre mice. Regions are labeled according to the mouse brain atlas (Franklin and Paxinos, 2001). Abbreviations: LPO, lateral preoptic area; BNST, bed nucleus of the stria terminalis; MPA, medial preoptic area; M2, secondary motor cortex; LHb, lateral habenular; LH, lateral hypothalamus; ZI, zona incerta; MM, medial mammillary nucleus, medial part; ZID, zona incerta, dorsal part; fr, fasciculus retroflexus; SC, superior colliculus; SNr, substantia nigra, reticular part; SNc, substantia nigra, compact part; IPN, interpeduncular nucleus; LDT, laterodorsal tegmental nucleus; CnF, cuneiform nucleus; PPT, pedunculopontine tegmental nucleus; PnO, pontine reticular nucleus, oral part; DR, dorsal raphe; MnR, median raphe nucleus; DRC, dorsal raphe, caudal part; DMTg, dorsomedial tegmental area.

To investigate the whole-brain input regions to RMTg GABAergic neurons, we cut and examined serial coronal sections after sufficient infection time for the three kinds of viruses. Representative sections from a VGAT-Cre mouse revealed that RMTg GABAergic neurons are innervated by whole-brain inputs. The dsRed-labeled afferent neurons were mainly located in the thalamus, hypothalamus, midbrain, and pons, such as the LHb, lateral hypothalamus (LH), zona incerta (ZI), superior colliculus (SC), DR, and laterodorsal tegmental nucleus (LDT) (Figure 3).

**FIGURE 4:**
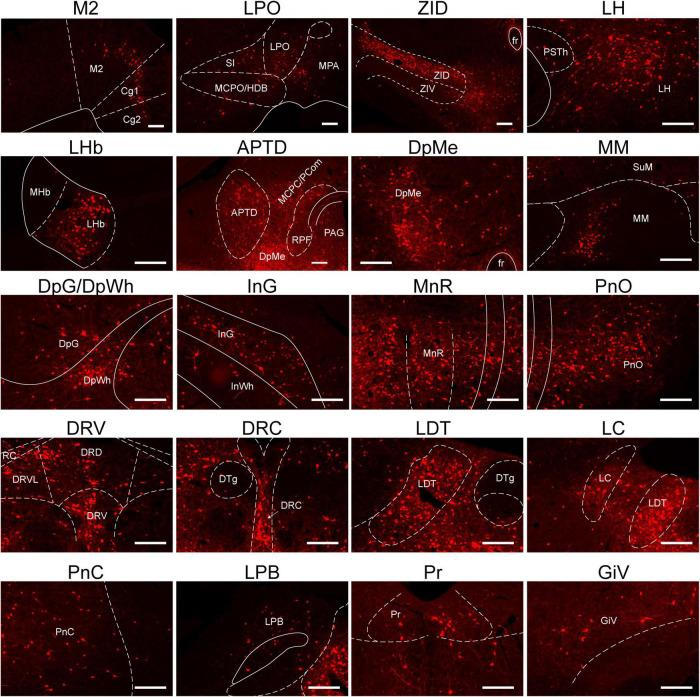
Representative nuclei with monosynaptic inputs to RMTg γ-aminobutyric acid-releasing (GABAergic) neurons. Abbreviations: M2, secondary motor cortex; Cg, cingulate cortex; LPO, lateral preoptic area; SI, substantia innominate; MCPO/HDB, magnocellular preoptic nucleus/nucleus of the horizontal limb of the diagonal band; MPA, medial preoptic area; ZID, zona incerta, dorsal part; ZIV, zona incerta, ventral part; fr, fasciculus retroflexus; PSTh, parasubthalamic nucleus; LH, lateral hypothalamus; LHb, lateral habenular; MHb, medial habenular; APTD, anterior pretectal nucleus, dorsal part; MCPC/PCom, magnocellular nucleus of the posterior commissure/nucleus of the posterior commissure; RPF, retroparafascicular nucleus; DpMe, deep mesencephalic nucleus; PAG, periaqueductal gray; SuM, supramammillary nucleus; MM, medial mammillary nucleus; DpG, deep gray layer of the superior colliculus; DpWh, deep white layer of the superior colliculus; InG, intermediate gray layer of the superior Colliculus; InWh, intermediate white layer of the superior colliculus; MnR, median raphe nucleus; PnO, pontine reticular nucleus, oral part; DRD, dorsal raphe nucleus, dorsal part; DRV, dorsal raphe, ventral part; DRVL, dorsal raphe nucleus, ventrolateral part; RC, raphe cap; DTg, dorsal tegmental nucleus; DRC, dorsal raphe, caudal part; LDT, laterodorsal tegmental nucleus; LC, locus coeruleus; PnC, pontine reticular nucleus, caudal part; LPB, lateral parabrachial nucleus; Pr, prepositus nucleus; GiV, gigantocellular reticular nucleus, ventral part. Scale bar, 200 μm.

The images in Figure 4 depict selected representative inputs innervating RMTg GABAergic neurons in detail. The representative input neurons are located in the nuclei of the following regions: the isocortex, specifically from the secondary motor cortex and cingulate cortex; lateral preoptic area (LPO); substantia innominata (SI); magnocellular preoptic nucleus/nucleus of the horizontal limb of the diagonal band; medial preoptic area (MPA); ZI, dorsal part (ZID); LH; LHb; anterior pretectal nucleus, dorsal part; magnocellular nucleus of the posterior commissure/nucleus of the posterior commissure; periaqueductal gray (PAG); retroparafascicular nucleus; deep mesencephalic nucleus (DpMe); medial mammillary nucleus; deep gray layer of the superior colliculus; deep white layer of the superior colliculus; intermediate gray layer of the superior colliculus; intermediate white layer of the superior colliculus; median raphe nucleus (MnR); pontine reticular nucleus (Pn), oral part; DR, dorsal part; DR, ventral part; DR, ventrolateral part; raphe cap; dorsal raphe, caudal part; LDT; locus coeruleus; pontine reticular nucleus, caudal part; lateral parabrachial nucleus; prepositus nucleus; gigantocellular reticular nucleus (Gi), ventral part (Figure 4).

### Immunostaining dsRed-Labeled Neurons With Several Neuronal Markers of Neurons Implicated in Sleep-Wake Regulation

Immunofluorescence assays showed that the monosynaptic inputs to RMTg GABAergic neurons colocalized with several markers of neurons implicated in diverse physiological functions, including the sleep-wake cycle. In the LH, the strongest presynaptic input nucleus to RMTg GABAergic neurons demonstrated in the present study, 12% of the dsRed-labeled neurons colocalized with hypocretin neurons which involved wakefulness. There were almost no dsRed-labeled neurons (2%) that colocalized with MCH-expressing neurons, which are known to promote rapid eye movement (REM) sleep (Scammell et al., 2017; Liu and Dan, 2019). Given that excitatory glutamatergic and inhibitory GABAergic neurons make up a large number of cell populations in the LH (Mickelsen et al., 2019), the dense monosynaptic projections to RMTg GABAergic neurons may result from glutamatergic or GABAergic neurons.

**FIGURE 5:**
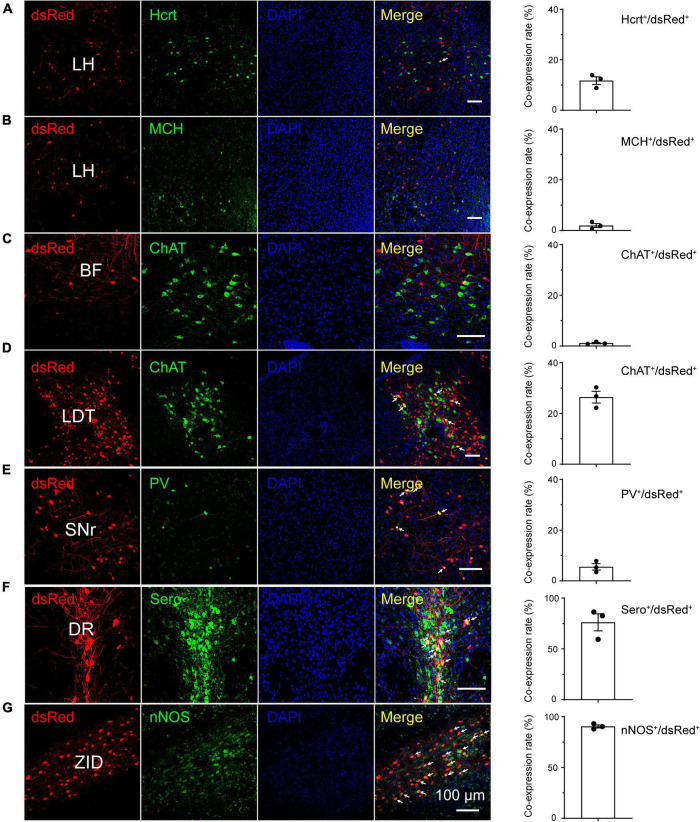
Immunostaining of typical nuclei innervating rostromedial tegmental area γ-aminobutyric acid-releasing (GABAergic) neurons with several markers of sleep-wake regulation. **(A–G)** Images showing that dsRed-labeled neurons colocalized with hypocretin (Hcrt) and melanin-concentrating hormone (MCH) in the lateral hypothalamus (LH) **(A,B)**, choline acetyltransferase (ChAT) in the basal forebrain (BF) and laterodorsal tegmental nucleus (LDT) **(C,D)**, parvalbumin (PV) in the substantia nigra, reticular part (SNr), **(E)** serotonin (Sero) in the dorsal raphe nucleus (DR) **(F)**, and neuronal nitric oxide synthase (nNOS) in the zona incerta, dorsal part (ZID) **(G)**. The merged neurons are pointed by arrows. Rightmost column, quantification of dsRed-labeled cells that co-expressed with specific cell type biomarkers. *n* = 3, each data point represents one experimental mouse. Abbreviation: DAPI, 4’, 6-diamidino-2-phenylindole.

In contrast, in the DR, most dsRed-labeled neurons, which accounted for 76%, colocalized with serotonergic neurons, which are known to regulate wakefulness and REM sleep. Although almost no dsRed-labeled neurons (1%) colocalized with ChAT in the basal forebrain (BF), many input neurons, which accounted for 26% in the LDT, were co-expressed with cholinergic neurons, which participate in REM sleep initiation (Scammell et al., 2017). Interestingly, a large number of dsRed-labeled neurons accounting for 90% in the ZID were colocalized with nNOS-expressing neurons. Unlikely, only 6% of the input neurons in the reticular part of the substantia nigra (SNr) colocalize with PV neurons (Figure 5).

**FIGURE 6:**
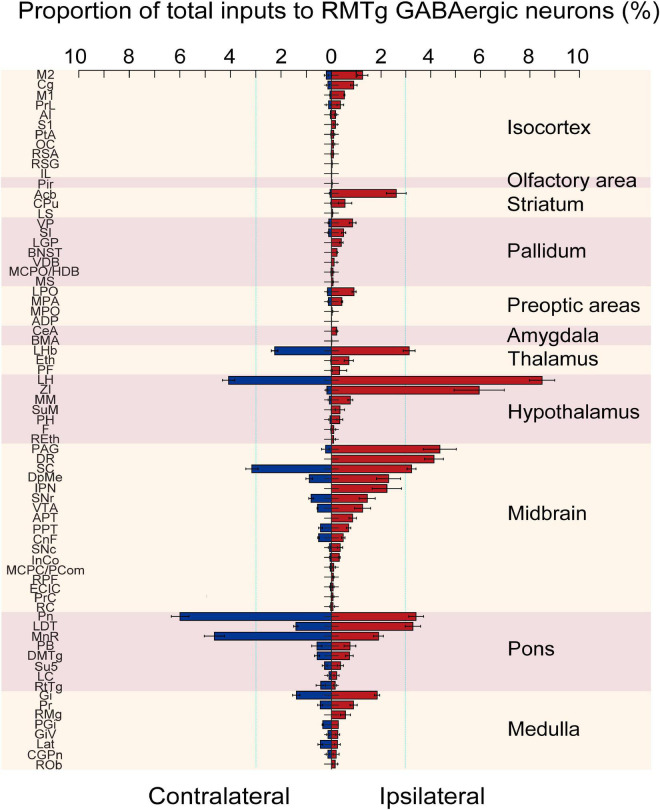
Statistical analysis of the whole-brain distribution of monosynaptic inputs to rostromedial tegmental area (RMTg) γ-aminobutyric acid-releasing (GABAergic) neurons in vesicular GABA transporter (VGAT)-Cre mice. The average proportion of dsRed-labeled neurons in each of the ipsilateral and contralateral brain regions with more than 0.1% of the total inputs to RMTg GABAergic neurons (*n* = 4). Brain areas are grouped into 11 structures. Left, contralateral inputs. Right, ipsilateral inputs. Abbreviations: M2, secondary motor cortex; Cg, cingulate cortex; M1, primary motor cortex; PrL, prelimbic cortex; AI, agranular insular cortex; S1, primary somatosensory cortex; PtA, parietal association cortex; OC, orbital cortex; RSA, retrosplenial agranular cortex; RSG, retrosplenial granular cortex; IL, infralimbic cortex; Pir, piriform cortex; Acb, accumbens nucleus; CPu, caudate putamen; LS, lateral septal nucleus; VP, ventral pallidum; SI, substantia innominate; LGP, lateral globus pallidus; BNST, bed nucleus of the striatum; VDB, nucleus of the vertical limb of the diagonal band; MCPO/HDB, magnocellular preoptic nucleus/nucleus of the horizontal limb of the diagonal band; MS, medial septal nucleus; LPO, lateral preoptic area; MPA, medial preoptic area; MPO, median preoptic nucleus; ADP, anterodorsal preoptic nucleus; CeA, central amygdaloid nucleus; BMA, basomedial amygdaloid nucleus; LHb, lateral habenular; Eth, ethmoid thalamic nucleus; PF, parafascicular thalamic nucleus; LH, lateral hypothalamus; ZI, zona incerta; MM, medial mammillary nucleus; SuM, supramammillary nucleus; PH, posterior hypothalamic area; F, nucleus of the fields of Forel; REth, retroethmoid nucleus; PAG, periaqueductal gray; DR, dorsal raphe nucleus; SC, superior colliculus; DpMe, deep mesencephalic nucleus; IPN, interpeduncular nucleus; SNr, substantia nigra, reticular part; VTA, ventral tegmental area; APT, anterior pretectal nucleus; PPT, pedunculopontine tegmental nucleus; CnF, cuneiform nucleus; SNc, substantia nigra, compact part; InCo, intercollicular nucleus; MCPC/PCom, magnocellular nucleus of the posterior commissure/nucleus of the posterior commissure; RPF, retroparafascicular nucleus; ECIC, external cortex of the inferior colliculus; PrC, precommissural nucleus; RC, raphe cap; Pn, pontine nuclei; LDT, laterodorsal tegmental nucleus; MnR, median raphe nucleus; PB, parabrachial nucleus; DMTg, dorsomedial tegmental area; Su5, supratrigeminal nucleus; LC, locus coeruleus; RtTg, reticulotegmental nucleus of the pons; Gi, gigantocellular reticular nucleus; Pr, prepositus nucleus; RMg, raphe magnus nucleus; PGi, paragigantocellular nucleus; GiV, gigantocellular reticular nucleus; Lat, lateral (dentate) cerebellar nucleus; CGPn, central gray of the pons; ROb, raphe obscurus nucleus. Data represent the mean ± standard error of the mean.

**FIGURE 7:**
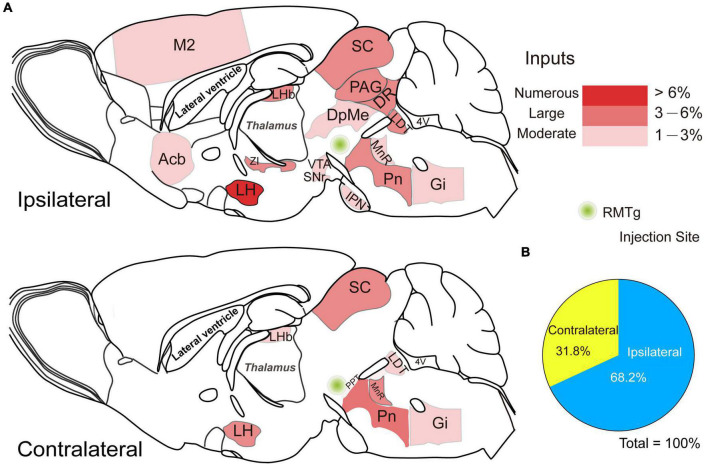
Schematic diagrams showing the distribution of the ipsilateral and contralateral monosynaptic inputs innervating rostromedial tegmental area (RMTg) γ-aminobutyric acid-releasing (GABAergic) neurons. **(A)** Sagittal sections for a schematic illustration of whole-brain inputs to RMTg GABAergic neurons in vesicular GABA transporter-Cre mice. **(B)** The proportion of ipsilateral and contralateral inputs to RMTg GABAergic neurons. Abbreviations: M2, secondary motor cortex; Acb, accumbens nucleus; LHb, lateral habenular; LH, lateral hypothalamus; ZI, zona incerta; PAG, periaqueductal gray; DR, dorsal raphe nucleus; SC, superior colliculus; DpMe, deep mesencephalic nucleus; IPN, interpeduncular nucleus; SNr, substantia nigra, reticular part; VTA, ventral tegmental area; PPT, pedunculopontine tegmental nucleus; Pn, pontine nuclei; LDT, laterodorsal tegmental nucleus; MnR, median raphe nucleus; Gi, gigantocellular reticular nucleus. Data represent the mean ± standard error of the mean.

### Statistics of Ipsilateral and Contralateral Input Neurons Innervating Rostromedial Tegmental Area GABAergic Neurons

After identifying the brain regions with monosynaptic inputs to RMTg GABAergic neurons, we performed a statistical analysis of the distribution of the input nuclei according to the percentage of the number of dsRed-labeled afferent neurons in each brain region to the total number of dsRed neurons in each of the whole brains (Figure 6, *n* = 4). We have identified 71 nuclei that had monosynaptic connections with RMTg GABAergic neurons, with each brain region having several dsRed-labeled neurons making up more than 0.1% of the total labeled neurons. Among these monosynaptic input neurons in the entire brain, 68.2% of the dsRed-labeled neurons were located on the ipsilateral side, while 31.8% were located on the contralateral side. The total afferent neurons originated from the following 11 brain structures: the isocortex, olfactory area, striatum, pallidum, preoptic areas, amygdala, thalamus, hypothalamus, midbrain, pons, and medulla.

Among the ipsilateral inputs, the secondary motor cortex (1.26 ± 0.21%) and cingulate cortex (0.90 ± 0.13%) comprised more dsRed-labeled neurons in the isocortex. In the striatum, the accumbens nucleus (Acb) provided the highest number of inputs (2.62 ± 0.40%). The first and second higher proportions of the total afferent neurons in the pallidum were in the ventral pallidum (VP) (0.85 ± 0.14%) and SI (0.49 ± 0.08%), respectively, while in the preoptic area, the LPO (0.91 ± 0.08%) and MPA (0.43 ± 0.03%) had more dsRed-labeled input cells than other regions. The LHb had the most input neurons in the thalamus (3.13 ± 0.24%). In the hypothalamus, the brain regions sending the first and second largest number of input neurons to RMTg GABAergic neurons were the LH (8.49 ± 0.51%) and ZI (5.95 ± 1.01%), respectively. In the midbrain, pons, and medulla, a lot of nuclei had stronger monosynaptic connections with proportions over 1% of total input neurons to RMTg GABAergic neurons, including the PAG (4.37 ± 0.67%), DR (4.13 ± 0.38%), SC (3.23 ± 0.18%), DpMe (2.30 ± 0.48%), interpeduncular nucleus (IPN) (2.23 ± 0.59%), SNr (1.44 ± 0.33%), VTA (1.26 ± 0.32%), Pn (3.41 ± 0.30%), LDT (3.28 ± 0.31%), MnR (1.90 ± 0.20%), and Gi (1.84 ± 0.10%).

In addition, we compared the proportions of the ipsilateral inputs with contralateral inputs. We found that RMTg GABAergic neurons were preferentially innervated by neurons on the ipsilateral side rather than their contralateral counterparts, but there were some exceptions. The contralateral Pn (5.99 ± 0.35%), MnR (4.64 ± 0.40%), reticulotegmental nucleus of the pons (RtTg, 0.43 ± 0.18%), paragigantocellular nucleus (PGi, 0.34 ± 0.03%), and lateral (dentate) cerebellar nucleus (Lat, 0.44 ± 0.10%) sent more monosynaptic projections to RMTg GABAergic neurons than their ipsilateral counterparts (Figure 6).

To compare the broad distribution of the input neurons more intuitively in the whole brain, we showed sagittal sections for schematic illustrations of the proportion of input neurons within each nucleus on either the ipsilateral or the contralateral side of the whole brain monosynaptic inputs to RMTg GABAergic neurons. It clearly showed that the numerous afferent neurons that accounted for over 6% of the total whole brain were located in the ipsilateral LH (Figure 7).

## Discussion

To understand how RMTg GABAergic neurons integrate information processing, it is essential to investigate the afferent connections that influence neuronal activity. Because of the non-specificity and inefficiency of conventional retrograde tracers, previous tracing studies did not represent comprehensive monosynaptic inputs to RMTg GABAergic neurons (Jhou et al., 2009b; Petzel et al., 2017). In the present study, we used a rabies-based system to label presynaptic afferents at the whole-brain level and quantified the number of input neurons. A total of 71 nuclei were identified that send presynaptic inputs to RMTg GABAergic neurons in the whole brain, such as the LH, LHb, Acb, VP, PAG, and DR. These enormous inputs arrived from the regions implicated in sleep/wake, motor, and mood regulation. Immunohistochemical staining showed that the input neurons colocalized with DR serotonergic neurons and LDT cholinergic neurons but not with BF cholinergic neurons. Several presynaptic inputs from the SNr are PV^+^ neurons. Notably, RMTg GABAergic neurons receive strong inputs from nNOS-expressing neurons in the ZID. Furthermore, the afferent pattern to RMTg GABAergic neurons was preferentially ipsilateral; however, there were some strong inputs from the contralateral side, such as the LHb, LH, SC, Pn, and MnR.

### Location of the Rostromedial Tegmental Area

The RMTg has been identified based on the Fos immunoreactivity following the administration of psychostimulants and the position of retrograde-labeled neurons stained by injection of tracers into the VTA (Geisler et al., 2008; Jhou et al., 2009a). In addition, by observing the presence and distribution of LHb input fibers within the RMTg through injection of transported viral vector encoding eGFP into the LHb, it was found that a part of the anterior tegmental nucleus and paramedian raphe were also included in the RMTg region (Quina et al., 2015). Recent reports on molecular markers further distinguished the RMTg from its surroundings. The transcription factor Foxp1 is highly expressed in the RMTg in adult rats (Lahti et al., 2016). When the retrograde tracer CTb was injected into the VTA of rats, about 83% of CTb-positive neurons expressed Foxp1 within the RMTg region, but only 4.5% of CTb-labeled cells expressed Foxp1 outside the RMTg. Those ratios in mice were 78% and 2%, respectively. Furthermore, the Foxp1-expressing neurons in the RMTg are GABAergic, as Foxp1 labeling colocalized strongly with VGAT expression within the RMTg (Smith et al., 2019). Therefore, Foxp1 could be recognized as a molecular marker to distinguish the RMTg from the adjacent area (Zhao et al., 2020; Jhou, 2021).

Here, we confirmed the location of the starter neurons in the RMTg through anatomical landmarks such as the IPN and superior cerebellar peduncle, as well as Foxp1 staining. The site of the starter neurons corresponded to prior mouse studies of the RMTg (Quina et al., 2015; Polter et al., 2018; Taylor et al., 2019; Sun et al., 2020).

### Comparison With Previous Retrograde Tracing Studies

Previous studies using classical non-specific retrograde tracers have shown that the strongest inputs to the RMTg originate from the LHb (Jhou et al., 2009b). Here, we used an RV-mediated retrograde tracing system in VGAT-Cre mice, which allowed specific labeling of presynaptic inputs innervating RMTg GABAergic neurons (Xu X. et al., 2020) and found that it is the LH, rather than the LHb, which provides the strongest dense presynaptic connections with RMTg GABAergic neurons. The proportion of inputs arising from the ipsilateral LH and LHb were 8.49 ± 0.51% and 3.13 ± 0.24%, respectively. In addition, the proportion of monosynaptic inputs in the ipsilateral ZI, PAG, DR, SC, Pn, and LDT was also higher than those in the LHb. Similarly, previous work showed that the VTA/SNc sent robust projections to the RMTg (Jhou et al., 2009b). However, our results illustrated that the VTA/SNc comprised fewer proportions of presynaptic inputs, which accounted for 1.26 ± 0.32% and 0.35 ± 0.11%, respectively. Unlike the medium inputs in the Pn revealed previously (Jhou et al., 2009b), the present study found large presynaptic input neurons in both the ipsilateral Pn (3.41 ± 0.30%) and contralateral Pn (5.99 ± 0.35%). Moreover, we found that 0.37 ± 0.14% and 0.75 ± 0.23% of the whole brain inputs to RMTg GABAergic neurons originated from the ipsilateral prelimbic cortex and PB, respectively, which is consistent with the findings in previous studies (Jhou et al., 2009b; Li et al., 2019a; Cruz et al., 2021).

In addition, we also identified several novel afferent nuclei to RMTg GABAergic neurons in mice, including the motor cortex, piriform cortex, median preoptic area, raphe areas, dorsomedial tegmental area, locus coeruleus, gigantocellular reticular nucleus, prepositus nucleus, lateral cerebellar nucleus, and central gray of the pons. These new findings will provide an anatomical basis for further functional studies of the RMTg.

Furthermore, we identified for the first time, that many direct monosynaptic afferents to RMTg GABAergic neurons also originate from the contralateral brain, such as the LHb, LH, SC, Pn, and MnR. The contralateral Pn and MnR had stronger projections than their ipsilateral counterparts. The function of this bilateral or preferential contralateral innervation pattern to the RMTg GABAergic neurons needs to be studied further. A recent study showed that unilateral optogenetic activation of indirect striatal projection neurons in the basal ganglia suppresses contraversive licking and promotes ipsiversive licking. This work illustrated that indirect striatal projection neurons implemented action selection via bilateral modulation of the activity of its downstream target SC (Lee and Sabatini, 2021). Cheng et al. (2021) found that the hepatosensitive region CA3 in the hippocampus receives direct projections from the bilateral LH area. In our study, the findings that the RMTg GABAergic neurons are bilaterally innervated by a large number of input neurons suggest that the RMTg may integrate diverse information from bilateral afferent regions such as the SC, LH, Pn, and LHb to regulate important functions.

### Functional Implications for Rostromedial Tegmental Area GABAergic Neurons in Sleep-Wake Regulation

We have found that RMTg GABAergic neurons receive strong inputs from many brain regions related to sleep-wake regulation. The LH is a functionally and anatomically diverse brain region that is known to mediate many physiological processes, including feeding, arousal, and energy balance. The LH MCH neurons regulate REM sleep while the orexinergic, glutamatergic, and GABAergic neurons in the LH promote arousal (Arrigoni et al., 2019). A large number of afferent neurons in the LH to RMTg GABAergic neurons are probably excitatory or inhibitory subtypes, which may promote non-REM (NREM) sleep by activation or disinhibition of RMTg GABAergic neurons, respectively (Yang S. R. et al., 2018). The ZI, which is adjacent to the LH, integrates sensory-motor information and projects heavily to major arousal centers of the thalamus and brainstem, suggesting that the ZI may influence sleep/wake states. It has been found that the transcription factor LHx6-expressing GABAergic neurons, which represent 45% of all GABAergic neurons in the ZI ventral part, promoted NREM and REM sleep (Liu et al., 2017). In the present study, the ZI was found to send the second largest number of inputs to RMTg GABAergic neurons and was colocalized with nNOS-expressing neurons. These nNOS-expressing neurons were co-expressed with glutamate and GABA in previous studies (Roger and Cadusseau, 1985; Ma et al., 1997; Power and Mitrofanis, 2001; Mitrofanis, 2005). Hence, it is worth investigating whether the prominent sleep promotion induced by the RMTg is controlled by the ZID. In the Acb, which had relatively dense projections to RMTg GABAergic neurons, the D1 receptor-expressing medium spiny neurons (D1-MSN) and D2-MSN have been shown to mediate wakefulness and NREM sleep, respectively (Oishi et al., 2017; Luo et al., 2018). The dopaminergic and glutamatergic neurons in the VTA have been revealed for wake-promotion and VTA GABAergic neurons for sleep promotion (Eban-Rothschild et al., 2016; Yu et al., 2019). The other relatively strong afferent inputs, such as the PAG, consist of REM-off GABAergic neurons, while the DR, LDT, and DpMe contain REM-on serotonergic, cholinergic, and GABAergic neurons, respectively (Weber et al., 2015; Liu and Dan, 2019). In addition, previous studies have suggested that other afferent inputs of the supramammillary nucleus (SuM), Pn, and Gi to RMTg GABAergic neurons are involved in REM sleep regulation (Verret et al., 2005; Luppi et al., 2006; Fuller et al., 2007; Renouard et al., 2015). These connections provide evidence that RMTg GABAergic neurons play important roles in the regulation of sleep/wake behavior.

### Functional Implications for Rostromedial Tegmental Area GABAergic Neurons in Motor Control

Previous studies have shown that SNr GABAergic neurons play a powerful role in suppressing movement (Hikosaka and Wurtz, 1985; Kravitz et al., 2010; Gerfen and Surmeier, 2011). There are heterogeneous subtypes within the SNr, with PV neurons being most active during locomotor states and glutamic acid decarboxylase 2 (GAD2) neurons being active during sleep and suppressed during periods of motor activities. The activation of SNr PV and GAD2 neurons terminates movement through transitions to quiet wakefulness and sleep, respectively (Liu et al., 2020). In our study, the findings of direct synaptic connections of RMTg GABAergic neurons with SNr PV neurons or with the motor cortex provide functional implications for RMTg GABAergic neurons in motor control (Levy et al., 2020). Although the midbrain dopaminergic system has long been found to play an important role in reward and prediction errors (Baik, 2013; Schultz, 2016), massive data have revealed that dopaminergic neurons are related to motor control. Dopaminergic neurons signal the onset of spontaneous movement in which movement-related signals are lost in a mouse model of Parkinson’s disease (Barter et al., 2015; Dodson et al., 2016; da Silva et al., 2018). Studies have shown that PAG neurons are crucial for gating and commanding the initiation of escape, such as running or jumping in situations of imminent threats (Lefler et al., 2020). In addition, the PAG controls all motor systems that generate vocalization, coughing, sneezing, vomiting, and respiration (Holstege, 2014). The DR constitutes a primary serotonergic input and modulates diverse functions, including those that are motor in nature (Ren et al., 2018). The SC is a sensorimotor structure that integrates visual and other sensory information to drive diverse behaviors, such as eye, head, or limb movements, through its vast outputs (Basso et al., 2021). Our data revealed ascending projections from the SNr, VTA/SNc, PAG, DR, and SC, suggesting that RMTg GABAergic neurons are implicated in the regulation of movement. This function was supported by the findings that rats showed vigorous locomotion induced by inhibition of the RMTg (Jhou et al., 2009a; Lavezzi et al., 2015).

### Implications for Rostromedial Tegmental Area GABAergic Neurons in Mood Control

The RMTg was first discovered by the observation that it was specifically activated by aversive stimuli such as foot shock and fasting (Jhou et al., 2009a). Similarly, in our study, we found that RMTg GABAergic neurons received monosynaptic inputs from the prelimbic cortex, PB, and LHb, which has been shown to drive triply dissociable RMTg responses to aversive cues, outcomes, and prediction errors, respectively (Li et al., 2019b). Moreover, the cingulate cortex was reported to be involved in pain-related aversion, while the PB is recognized as a sensory hub for pain and aversive behaviors (Hayes and Northoff, 2011; Chiang et al., 2019; Meda et al., 2019).

Depression is a common mental health disorder. Loss of motivation is one of the main characteristics of depression, which is manifested by impairments in reward-seeking behavior and escape from punishment (Salamone and Correa, 2012; Jean-Richard-Dit-Bressel et al., 2018). The generation of motivation is closely associated with the brain’s reward centers, including the VTA and DR (Ren et al., 2018). Moreover, Fernandez et al. (2018) found that chronic social defeat stress, a preclinical paradigm of depression, causes marked hyperactivity of LDT cholinergic neurons. Chemogenetic inhibition of these neurons prevents depression-like behaviors. Accumulating evidence has shown that overactivity in the LHb is crucial for driving depression-like behaviors (Yang et al., 2008; Yang Y. et al., 2018). Optogenetic activation of the projection from the LHb resulted in an increase in immobility in forced swimming tests and loss of motivation to eat (Proulx et al., 2018). Similar studies have shown that the RMTg plays an important role in the expression of anhedonia and depression-like behaviors induced by stress and withdrawal from chronic alcohol consumption in rats (Elmer et al., 2019; Fu et al., 2019). In a recent study, Markovic et al. (2021) found that pain increased RMTg inhibitory tone onto VTA dopaminergic neurons, making them less excitable and finally leading to anhedonia-like behavior.

Regarding the presynaptic inputs to RMTg GABAergic neurons, the Acb and VP have been known as key brain regions for regulating drug addiction (Xu L. et al., 2020; Kupchik and Prasad, 2021). The results of the existing research suggest that the activities of RMTg neurons are profoundly influenced by addictive drugs, including opioids (Barrot, 2015) and alcohol (Fu et al., 2019; Glover et al., 2019).

In this study, we found that RMTg GABAergic neurons received monosynaptic innervation of multiple important emotional regulatory neural circuits, such as the VTA, DR, LDT, LHB, Acb, and VP, suggesting that the RMTg may be involved in mood regulation.

In summary, for the first time, we mapped and quantified the afferents of RMTg GABAergic neurons and found that they receive extensive ascending projections. Most of them preferentially arise from the ipsilateral rather than the contralateral counterpart. Moreover, we found several novel afferent nuclei and identified several neuronal types innervating RMTg GABAergic neurons. Our data suggest that RMTg GABAergic neurons have diverse physiological and pathological functions, particularly those involved in sleep/wake, motor, and mood regulation. Our results provide anatomical evidence for the elucidation of the roles of RMTg GABAergic neurons in modulating multiple behaviors.

It is worth noting that in VGAT-Cre mice, *in vitro* electrophysiological recording showed that the amplitude of inhibitory postsynaptic currents of VTA dopaminergic neurons evoked by photostimulation of RMTg afferents was slightly reduced by glycine receptor antagonist strychnine, whereas they were completely blocked by GABA_*A*_ receptor antagonist bicuculline, indicating the inhibitory inputs from RMTg to VTA dopaminergic neurons are dominantly by GABA release (Polter et al., 2018). These results suggest that the targeted neurons using VGAT-Cre mice mainly release GABA and possibly co-release glycine.

## Data Availability Statement

The raw data supporting the conclusions of this article will be made available by the authors, without undue reservation.

## Ethics Statement

The animal study was reviewed and approved by the Committee on the Ethics of Animal Experiments of the School of Basic Medical Sciences, Fudan University.

## Author Contributions

S-RY contributed to the conception and design of the study, wrote and edited the manuscript, and supervised the study. Y-NZ acquired the database, performed the data analysis, and wrote the manuscript. YZ and S-YT helped with the data acquisition. S-RY, W-MQ, and Z-LH provided funding support. W-MQ gave comments. All authors contributed to manuscript revision, read, and approved the submitted version.

## Conflict of Interest

The authors declare that the research was conducted in the absence of any commercial or financial relationships that could be construed as a potential conflict of interest.

## Publisher’s Note

All claims expressed in this article are solely those of the authors and do not necessarily represent those of their affiliated organizations, or those of the publisher, the editors and the reviewers. Any product that may be evaluated in this article, or claim that may be made by its manufacturer, is not guaranteed or endorsed by the publisher.
